# The role of complement factor H in gestational diabetes mellitus and pregnancy

**DOI:** 10.1186/s12884-021-04031-w

**Published:** 2021-08-17

**Authors:** Junxian Li, Ying Shen, Hairong Tian, Shuting Xie, Ye Ji, Ziyun Li, Junxi Lu, Huijuan Lu, Bo Liu, Fang Liu

**Affiliations:** 1 Department of Endocrinology & Metabolism, Shanghai Jiao-Tong University Affiliated Sixth People’s Hospital, Shanghai Key Laboratory of Diabetes, Shanghai Clinical Medical Center of Diabetes, Shanghai Key Clinical Center of Metabolic Diseases, Shanghai Institute for Diabetes, Shanghai, 200233 China; 2 Department of Endocrinology & Metabolism, The Affiliated Jiangsu Shengze Hospital of Nanjing Medical University, Suzhou, 215228 China; 3 Department of Endocrinology and Metabolism, Jin Shan Branch of Shanghai Sixth People’s Hospital, Shanghai, 201599 China; 4 Department of Endocrinology and Metabolism, Shanghai General Hospital, Shanghai Jiao Tong University School of Medicine, Shanghai, 200080 China

**Keywords:** Complement factor H, Gestational diabetes mellitus, Pregnancy

## Abstract

**Background:**

Complement factor H (CFH) has been found to be associated with insulin resistance. This study assessed the correlation between CFH and other clinical parameters, and determined whether CFH played a role in gestational diabetes mellitus (GDM) and adverse pregnancy outcomes.

**Methods:**

A total of 397 pregnant women were included for analysis in this nested case-control study. Clinical parameters and serum were collected within the 11-17th gestational age at the first prenatal visit. At 24–28 weeks of gestation, a 75 g oral glucose tolerance test was performed and subjects were divided into a GDM (*n* = 80) and a non-GDM control group (*n* = 317). The delivery data were also followed. The serum CFH level was assayed by ELISA.

**Results:**

CFH was higher in GDM than in non-GDM controls (280.02 [58.60] vs. 264.20 [68.77]; *P* = 0.014). CFH level was moderately associated with pre-pregnancy body mass index (BMI), BMI and total triglycerides (TG), and slightly associated with gestational age, low density lipoprotein cholesterol (LDL-C), total cholesterol (TC) in GDM and non-GDM (all *P* <  0.05). Moreover, CFH level was moderately correlated with alkaline phosphatase (ALP) and slightly correlated with age, uric acid (UA) and total bilirubin (TB) in non-GDM (all *P* <  0.05). After adjustment for clinical confounding factors, BMI, TG, gestational age, ALP, TB, age and UA were independent risk factors for log_10_ CFH levels (all *P* <  0.05) in all subjects. In addition, overweight or obese pregnant women, women with hypertriglyceridemia and women in the second trimester had significantly higher CFH levels than normal weight and underweight group (*P* <  0.001), the non-hypertriglyceridemia group (*P* <  0.001) and women in the first trimester group (*P <* 0.05) in all pregnant women respectively. Following binary logistic regression, CFH was not independently associated with GDM and related pregnant outcomes.

**Conclusions:**

The CFH in 11-17th weeks of gestation might be affected by many factors, including BMI, TG, gestational age, ALP, TB, age and UA. CFH was not an independent risk factor for GDM and avderse pregnancy outcomes.

**Supplementary Information:**

The online version contains supplementary material available at 10.1186/s12884-021-04031-w.

## Background

Gestational diabetes mellitus (GDM) is a condition in which glucose intolerance appears anytime during pregnancy leading to rise in blood glucose levels. The incidence of GDM varies according to diagnostic criteria, geographic regions and race/ethnicity. In addition, its incidence is expected to increase in the future [1–3].

The pathogenesis of GDM includes insulin resistance and insufficient insulin secretion, but the specific mechanism remains unclear [3]. Gestational hyperglycemia has serious adverse consequences on pregnant mothers, the developing fetus and neonates, including cesarean section, macrosomia, and premature rupture of membranes (PROM). Long-term consequences from GDM include development of cardiovascular disease (CVD) and type 2 diabetes mellitus (T2DM) in both mothers and offsprings and additionally obesity in the offsprings [3, 4]. Therefore, a coordinated study on the disease pathogenesis of GDM and predictive biomarkers is of great significance.

Previous researches found that inflammation might play a key role in the pathogenesis of GDM and inflammatory cytokines were predictive biomarkers of GDM [5]. For instance, Ueland et al. found that the macrophage marker sCD163 increased at 14-16th weeks of gestation [6], and Ozgu-Erdinc et al. reported that C-reactive protein (CRP) increased within 11-14th weeks of gestation [7], and both inflammatory markers were independently associated with GDM. On the contrary, other researchers found that combination of clinical factors and biomarkers such as TNF-alpha and high sensitivity-CRP did not show significant improvement in the prediction of GDM [8, 9].

The complement system is an important part of innate immunity, and its activation occurs through three distinct pathways: the classical pathway, the lectin pathway and the alternative pathway [10]. Human complement factor H (CFH) is a soluble complement system inhibitor and can protect cells and tissues from unexpected complement system-mediated damage [11]. The gene that encodes CFH is located on chromosome 1q31.3 and is mainly expressed by the liver [12, 13], and other cell types including endothelial cells [14], retinal pigment epithelial cells [15], and adipocytes [16, 17]. CFH levels in the plasma varied widely from 116 to 562 μg/ml depending on genetic and environmental factors [11, 18], and might even increase in pregnant women [19].

It has been suggested that CFH was associated with obesity and metabolic disorders. Moreno-Navarrete et al. found that the CFH level significantly increased in patients with altered glucose tolerance and T2DM, and plasma CFH levels were negatively associated with insulin sensitivity [16, 20]. It was considered that attenuated insulin sensitivity represents the main pathogenic mechanism in GDM, and thus CFH might be related to GDM development.

Recently, Shen et al. revealed that complement system-associated proteins, including CFH, changed significantly in GDM at 12-14th gestational age as measured by proteomic analysis [21]. Therefore, the role of CFH in GDM patients requires further study.

Thus, the aim of this study was to assess the correlation between CFH and other clinical parameters in Chinese pregnant women, and to determine whether CFH played a role in GDM and avderse pregnancy outcomes.

## Methods

### Study population

It was a nested case-control study. Pregnant women were recruited in a prospective cohort and drawn blood samples at the first prenatal visit (< 24th gestational age). Inclusion criteria included the following: the first prenatal visit that was less than 24 weeks gestation; do not smoke or consume alcohol; no pre-existing medical disorders including diabetes and acute or chronic inflammation. A total of 607 women who met the inclusion criteria were recruited at the first prenatal visit. At 24–28th weeks of gestation, all women experienced the 75-g oral glucose tolerance test (75-g OGTT) and GDM was diagnosed if one of the following criteria was met or exceeded: 0 h glucose ≥5.1 mmol/L, 1 h glucose ≥10 mmol/L, and 2 h glucose ≥8.5 mmol/L [22]. Clinical and biochemical data from the first prenatal visit to delivery were collected at the Department of Obstetrics and Gynecology and the Department of Endocrinology and Metabolism of the Jin Shan Branch of Shanghai Sixth People’s Hospital, from February 2017 to April 2019. Subsequently, a total of 210 were excluded due to preconception diabetes (*n* = 4), twin pregnancy (n = 4) and incomplete clinical or measurement data (*n* = 202). The final number of women included for analysis was 397, and those women’s first prenatal visits were within the 11-17th gestational age.

### Data and serum sample collection

All pregnant women who met the inclusion criteria completed questionnaires (Additional file 1) that collected general background information including age, last menstrual period, reproductive history, and family history of diabetes at the first prenatal visit. Moreover, height, weight, and systolic and diastolic blood pressure were recorded on a standardized form by the physician during the examination. Pre-pregnancy body mass index (BMI) was calculated as pre-pregnancy body weight (in kg)/height^2^ (in m^2^). BMI was calculated at point of first prenatal visit and was calculated as body weight (in kg)/height^2^ (in m^2^). Each participant was drawn 3 ml venous blood following one night of fasting at the first prenatal visit, and serum samples were obtained aseptically by centrifugation at 3500 rpm for 15 min, which were then frozen at − 80 °C until being used [23]. Macrosomia was defined as birth weight ≥ 4000 g. Premature rupture of membrane (PROM) was defined as rupture of membranes before the onset of labour [24]. Estimated blood loss at delivery was defined as volume of blood loss from women during delivery within first 24 h after birth, and was calculated by the following ways: gauzes and pads with blood were weighed and an equivalent volume was estimated; blood volume in the suction bottle was measured.

### Laboratory measurements

Plasma glucose values were measured by the glucose oxidase method. HbA1c was determined by high-pressure liquid chromatography. Glycated serum albumin (GA) was tested by the liquid enzymatic assay. Other biochemical indices evaluating hepatic and renal functions such as aspartate aminotransferase (AST), alanine aminotransferase (ALT), total bilirubin (TB), γ-glutamyltransferase (γ-GT), alkaline phosphatase (ALP), creatinine (Cr), blood urea nitrogen (BUN), and uric acid (UA) were performed by enzymatic methods. Serum lipids including total triglycerides (TG), total cholesterol (TC) and low density lipoprotein cholesterol (LDL-C) were also tested by enzymatic methods. Serum albumin (ALB) was measured by the Bromocresol Green (BCG) dye-binding method. Then serum CFH concentrations were measured in duplicate by using MicroVue Factor H EIA kits (Quidel Corporation, USA) according to the manufacturer’s instructions, and the detectable quantitation range was 4.64–521 ng/ml. The intra- and inter-assay coefficients of variation (CV) were less than 10%. The control values were within the control ranges.

### Statistical analysis

Data are expressed as median (interquartile range, [IQR]) for continuous variables with non-normal distribution, mean ± standard deviation (SD) for continuous variables with normal distribution, and percentage (%) for categorical variables. Differences between groups were evaluated with the Chi-square test for categorical variables and the Student’s t-test or Mann-Whitney U test for continuous variables. The correlation between CFH and other characteristics at the first prenatal visit was evaluated with the Spearman’s rank correlation and multiple stepwise linear regression analysis. Binary logistic regression analysis was performed to evaluate the odds ratios (OR) and 95% confidence intervals (CIs) in multivariable analysis. All statistical analyses were measured by SPSS version 26.0 (SPSS Inc., Chicago, IL, USA). A two-sided alpha value of *P* <  0.05 was considered statistically significant.

## Results

### Subject characteristics

In this nested case-control study, 397 women completed the study and were assigned into a GDM (*n* = 80) and a non-GDM group (*n* = 317) based on the 75 g OGTT results at 24-28th gestational age. The clinical and biochemical characteristics of both are shown in Table 1. There were significant differences in terms of age, pre-pregnancy BMI, BMI, FPG, HbA1c, ALT, UA, TG, TC, LDL-C and CFH (all at *P* <  0.05) between the GDM and non-GDM controls. After comparison of pregnant outcomes, the incidence of macrosomia (%) was significantly higher in GDM than in non-GDM controls (*P* <  0.05). However the incidence of caesarean section (%), PROM (%), fetal distress (%), and other outcomes such as gestational age at delivery, estimated blood loss at delivery, and the Apgar score showed no differences between GDM and non-GDM controls.

**Table 1:** Comparison of the clinical characteristics of pregnant women with and without gestational diabetes mellitus (GDM)

	GDM (*n* = 80)	Non-GDM (*n* = 317)	
Parameters^a^	Median (IQR) or mean ± SD or %	Median (IQR) or mean ± SD or %	*P* value^b^
Gestational age at the first prenatal visit, week	14.00 (2.75)	13.00 (3.00)	0.292^†^
Age, years	29.00 (5.00)	27.00 (5.00)	0.012^†^
Pre-pregnancy BMI, kg/m^2^	21.70 (4.33)	20.80 (3.65)	< 0.001^†^
BMI, kg/m^2^	21.98 (4.93)	20.90 (3.75)	< 0.001^†^
SBP, mmHg	118.00 (16.75)	115.00 (14.00)	0.215^†^
DBP, mmHg	75.00 (11.00)	74.00 (12.50)	0.502^†^
Family History of diabetes, %	10	4.7	0.125^#^
GA, %	12.08 ± 1.91	11.92 ± 1.55	0.422^*^
FPG, mmol/L	4.85 (0.60)	4.70 (0.50)	< 0.001^†^
HbA1c, %	5.20 (0.40)	5.10 (0.30)	< 0.001^†^
ALT, units/L	14.50 (16.50)	12.00 (11.00)	0.024^†^
AST, units/L	16.00 (7.60)	16.00 (5.30)	0.940^†^
γ-GT, units/L	11.50 (8.30)	11.00 (8.30)	0.425^†^
ALP, units/L	45.00 (15.75)	46.00 (14.00)	0.488^†^
TB, μmol/L	8.20 (4.58)	7.90 (3.75)	0.539^†^
ALB, g/L	42.10 (3.05)	42.100 (3.65)	0.872^†^
BUN, mmol/L	2.80 (0.90)	2.70 (0.80)	0.458^†^
Cr, μmol/L	44.00 (8.00)	44.00 (8.00)	0.197^†^
UA, μmol/L	220.50 (62.00)	204.00 (60.00)	0.004^†^
TG, mmol/L	1.49 (0.79)	1.295 (0.65)	0.007^†^
TC, mmol/L	4.62 ± 0.79	4.42 ± 0.78	0.042^*^
LDL-C, mmol/L	2.52 ± 0.67	2.36 ± 0.66	0.051^*^
CFH, μg/ml	280.02 (58.60)	264.20 (68.77)	0.014^†^
GDM screening 75 g OGTT			
Gestational age at 75 g OGTT, week	25.00 (2.00)	25.00 (1.00)	0.777^†^
Glucose 0 h, mmol/L	5.15 (0.70)	4.50 (0.50)	< 0.001^†^
Glucose 1 h, mmol/L	9.79 ± 1.82	7.24 ± 1.39	< 0.001^*^
Glucose 2 h, mmol/L	7.95 (2.15)	6.31 (1.56)	< 0.001^†^
Pregnancy outcomes			
Gestational age at delivery, week	39.00 (1.00)	39.00 (1.00)	0.100^†^
Caesarean section, %	48.8	42.3	0.296^#^
PROM, %	11.3	11.4	0.979^#^
Estimated blood loss at delivery, ml	300.00 (90.00)	300.00 (100.00)	0.71^†^
Macrosomia, %	11.0	4.4	0.038^#^
Apgar score	10.00 (0.00)	10.00 (0.00)	0.291^†^

*Abbreviations*: *GDM* gestational diabetes mellitus; *BMI* body mass index; *SBP* systolic blood pressure; *DBP* diastolic blood pressure; *GA* Glycated serum albumin; *FPG* fasting plasma glucose; *HbA1c* glycosylated hemoglobin A1c; *ALT* alanine aminotransferase; *AST* aspartate aminotransferase; *γ-GT* γ-glutamyltransferase; *ALP* alkaline phosphatase; *TB* total bilirubin; *ALB* albumin; *BUN* blood urea nitrogen; *Cr* creatinine; *UA* uric acid; *TC* total cholesterol; *TG* total triglycerides; *LDL-C* low-density lipoprotein cholesterol; *CFH* complement factor H; *PROM* premature rupture of membrane

a. Data are expressed as median (interquartile range, [IQR]) for continuous variables with non-normal distribution, mean ± standard deviation (SD) for continuous variables with normal distribution, and percentage (%) for categorical variables

b.^*^Derived from Student’s t-test. ^†^Derived from Mann-Whitney U test. ^#^Derived from Chi-square test

### The association between CFH and other clinical and biochemical characteristics

To assess the relationship between CFH and other parameters, the Spearman’s correlation analysis was used to derive a correlation coefficient (r). The result showed that CFH was found to be significantly moderately positively (0.3 ≤ r <  0.5) associated with pre-pregnancy BMI, BMI and TG, and significantly slightly positively (r <  0.3) associated with gestational age, LDL-C, TC in GDM, non-GDM (Table 2). In addition, this study showed that the CFH level was moderately correlated with ALP and slightly correlated with age, UA and TB (all *P* <  0.05) in non-GDM (Table 2).

**Table 2:** Correlation between complement factor H (CFH) and other variables in the first prenatal visit

	CFH (μg/ml)			
	GDM (*n* = 80)		Non-GDM (*n* = 317)	
	r	*P*	r	*P*
Gestational age at the first prenatal visit, week	0.235	0.036	0.141	0.012
Age, years	0.076	0.504	0.199	< 0.001
Pre-pregnancy BMI, kg/m^2^	0.319	0.004	0.344	< 0.001
BMI, kg/m^2^	0.355	0.001	0.366	0.000
SBP, mmHg	0.214	0.057	0.104	0.064
DBP, mmHg	0.167	0.140	0.099	0.079
Family history of diabetes, %	0.000	1.000	0.164	0.003
GA, %	0.072	0.524	−0.083	0.139
FPG, mmol/L	0.003	0.979	0.095	0.091
HbA1c, %	0.191	0.089	0.174	0.002
ALT, units/L	0.083	0.464	0.035	0.530
AST, units/L	0.103	0.365	0.018	0.750
γ-GT, units/L	0.070	0.537	0.109	0.052
ALP, units/L	0.127	0.263	0.392	< 0.001
TB, umol/L	−0.142	0.208	−0.218	< 0.001
ALB, g/L	−0.037	0.747	−0.081	0.148
BUN, mmol/L	0.005	0.966	−0.093	0.101
Cr, μmol/L	−0.078	0.491	0.017	0.766
UA, μmol/L	0.142	0.209	0.231	< 0.001
TG, mmol/L	0.312	0.005	0.319	< 0.001
TC, mmol/L	0.235	0.038	0.189	0.001
LDL-C, mmol/L	0.241	0.034	0.189	0.001

*Abbreviations*: *CFH* complement factor H; *GDM* gestational diabetes mellitus; *r* correlation coefficient; *BMI* body mass index; *SBP* systolic blood pressure; *DBP* diastolic blood pressure; *GA* Glycated serum albumin; *FPG* fasting plasma glucose; *HbA1c* glycosylated hemoglobin A1c; *ALT* alanine aminotransferase; *AST* aspartate aminotransferase; *γ-GT* γ-glutamyltransferase; *ALP* alkaline phosphatase; *TB* total bilirubin; *ALB* albumin; *BUN* blood urea nitrogen; *Cr* creatinine; *UA* uric acid; *TG* total triglycerides; *TC* total cholesterol; *LDL-C* low-density lipoprotein cholesterol; *TB* total bilirubin. Data were derived from Spearman’s rank correlation analysis

**Fig. 1:**
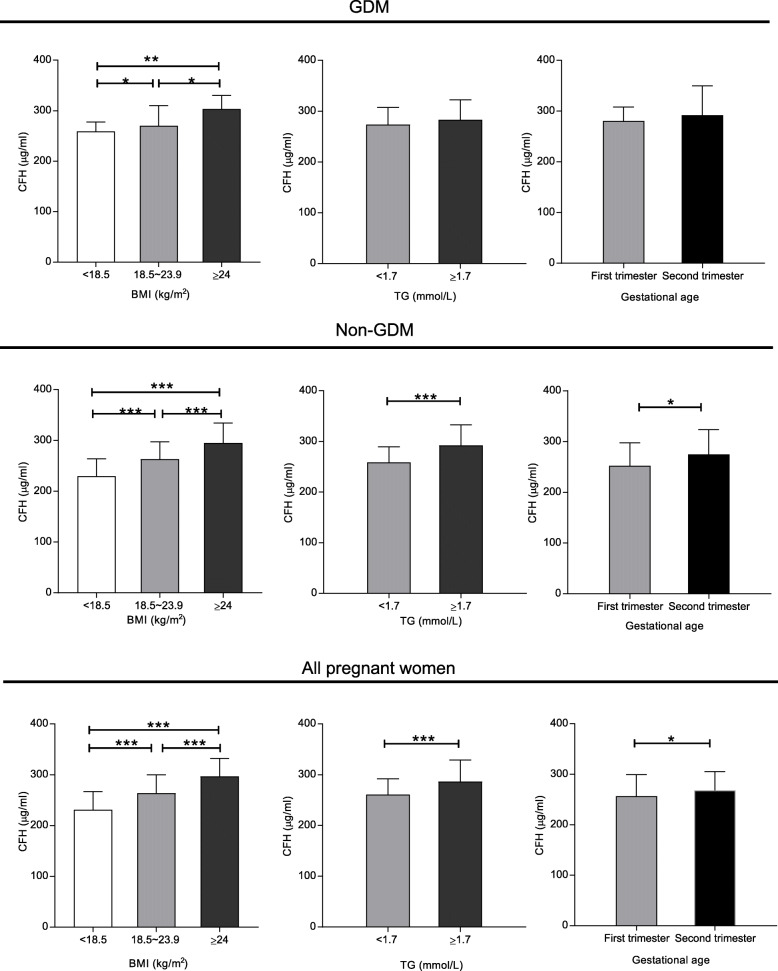
The CFH increased with body mass index (BMI), total triglyceride (TG) and gestational age. The CFH levels are different in three BMI categories, two TG categories and trimesters of pregnancy. CFH, complement factor H; GDM, gestational diabetes mellitus. The plot shows the median with the interquartile range. The *P* value is compared between two categories.*P < 0.05, **P < 0.01, *** *P* < 0.001

The pregnant women were divided into three categories by BMI: an underweight category (BMI < 18.5 kg/m^2^), a normal weight category (18.5 < BMI < 23.9 kg/m^2^), and an overweight or obese category (BMI ≥ 24 kg/m^2^). The result showed that overweight or obese pregnant women had significantly higher levels of CFH as compared to normal and underweight pregnant women in GDM (303.45 [58.39] vs. 270.29 [61.63] vs. 259.00 [54.13], respectively, *P* < 0.01), in non-GDM controls (294.93 [68.32] vs. 263.13 [58.63] vs. 229.55 [49.70], respectively, *P* < 0.001) and in all pregnant women (296.49 [65.11] vs. 263.43 [59.61] vs. 230.93 [51.36], respectively, *P* < 0.001) (Fig.1). Hypertriglyceridemia is a lipid metabolism disorder, so the subjects were divided into two categories by TG: a non-hypertriglyceridemia category (TG < 1.7 mmol/L) and a hypertriglyceridemia category (TG ≥ 1.7 mmol/L). Participants with hypertriglyceridemia had significantly higher CFH levels than non-hypertriglyceridemia participants in non-GDM (290.23 [85.29] vs. 259.58 [59.26], respectively, *P* < 0.001) and in all pregnant women (291.55 [75.46] vs. 260.60 [60.28], respectively, *P* < 0.001) (Fig. 1). In addition, the CFH level was also significantly higher in the second trimester (13 ~ 28 gestational age) than in the first trimester (0 ~ 12 gestational age) in non-GDM (265.25 [64.09] vs. 252.19 [71.24], respectively, *P* = 0.037) and in all pregnant women (268.04 [60.96] vs. 256.81 [71.68], respectively, *P* = 0.019) (Fig. 1).

### Multiple stepwise linear regression analysis of possible independent risk factors of log_10_ CFH

To determine which factors were independently associated with serum CFH levels, the multiple stepwise linear regression was performed in all subjects. Clinical parameters including gestational age, age, BMI, SBP, DBP, family history of diabetes, FPG, ALT, AST, γ-GT, ALP, TB, ALB, BUN, UA, TG, TC and LDL-C were included in analysis of multiple stepwise linear regression. This analysis revealed that BMI (Standardized Coefficients Beta [β] = 0.230, *P* < 0.001), TG (β = 0.130, *P* = 0.011), gestational age (β = 0.138, *P* = 0.004), ALP (β = 0.197, *P* < 0.001),TB (β = − 0.174, *P* < 0.001), age (β = 0.111, *P* = 0.020), and UA (β = 0.106, *P* = 0.027) were independent risk factors for serum log_10_ CFH levels in all pregnant women.

### Binary logistic regression analysis of factors affecting GDM and adverse pregnancy outcomes

To determine whether CFH was independently associated with GDM and undesirable pregnancy outcomes or not, binary logistic regression was performed (Table 3). However, no significantly independent association was found between serum CFH and GDM and avderse pregnancy outcomes. In addition, BMI was independently associated with GDM (OR, 1.090; 95% CI, 1.005–1.183; *P* = 0.037). In the macrosomia subgroup, BMI was also an independent risk factor for macrosomia development (OR, 1.203; 95% CI, 1.065–1.360; *P* = 0.003). In the caesarean subgroup, age (OR, 1.127; 95% CI, 1.068–1.190; *P* < 0.001), BMI (OR, 1.092; 95% CI, 1.011–1.179; *P* = 0.026) and ALT (OR, 1.024; 95% CI, 1.006–1.043; *P* = 0.008) were independent risk factors. We did not identify any factor that was significantly correlated with PROM in this study.

**Table 3:** Binary logistic regression analysis of factors affecting gestational diabetes mellitus (GDM) and adverse pregnancy outcomes

	GDM	Macrosomia	Caesarean section	PROM
	OR (95% CI)	OR (95% CI)	OR (95% CI)	OR (95% CI)
CFH	1.002 (0.996–1.007)	0.993 (0.982–1.003)	0.997 (0.992–1.002)	0.997 (0.990–1.005)
Age	1.042 (0.981–1.107)	0.966 (0.868–1.074)	1.127 (1.068–1.190)***	1.033 (0.959–1.113)
BMI	1.090 (1.005–1.183)*	1.203 (1.065–1.360)**	1.092 (1.011–1.179)*	0.994 (0.890–1.110)
Family history of diabetes	1.457 (0.551–3.853)	0.385 (0.046–3.233)	2.041 (0.756–5.510)	0.708 (0.150–3.336)
ALT	1.008 (0.991–1.026)	1.019 (0.993–1.045)	1.024 (1.006–1.043)**	1.002 (0.979–1.026)
ALP	0.995 (0.971–1.020)	1.008 (0.968–1.050)	0.995 (0.974–1.017)	1.009 (0.978–1.041)
TB	1.035 (0.946–1.133)	0.842 (0.706–1.004)	1.045 (0.968–1.129)	0.978 (0.871–1.099)
UA	1.003 (0.997–1.009)	1.000 (0.990–1.009)	1.002 (0.997–1.007)	1.000 (0.993–1.008)
TG	1.211 (0.783–1.874)	1.312 (0.647–2.660)	1.021 (0.684–1.523)	0.705 (0.373–1.332)
TC	1.123 (0.789–1.599)	0.980 (0.545–1.764)	1.074 (0.794–1.451)	1.429 (0.926–2.206)

*Abbreviations*: *GDM* gestational diabetes mellitus; *PROM* premature rupture of the membrane; *OR* odds ratio; *95% CI* 95% confidence interval; *CFH* complement factor H; *BMI* body mass index; *ALT* alanine aminotransferase; *ALP* alkaline phosphatase; *TB* total bilirubin; *UA* uric acid; *TG* total triglycerides; *TC* total cholesterol. Data were derived from binary logistic regression. *P < 0.05, **P < 0.01, and ***P < 0.001

## Discussion

The CFH was found to be higher in GDM as compared with non-GDM controls in Chinese women. In addition, the CFH was independently associated with BMI, TG, gestational age, ALP, age, TB and UA in all subjects. Following binary logistic regression, CFH was not independently associated with GDM and pregnancy outcomes. There are some strengths in this study. First, the research tested CFH firstly in pregnant women whose prenatal and delivery clinical data were also followed. Second, this study found some clinical parameters that might independently affect CFH levels and the complement system activity during pregnancy.

Some results of this study were generally consistent with previous work. This study and that of others found that CFH was positive associated with BMI [16, 25, 26], and fasting TG [16]. It was reported [16, 25] that alternative complement activation was associated with elevated BMI and the synthesis of TG in adipocytes, because C3, C3a, and other alternative pathway components were all associated with BMI [25, 26], and the C3a degradation product C3a des-Arg could stimulate the synthesis of TG in adipocytes further [27]. Since CFH is a complement alternative pathway inhibitor, it might represent a compensatory increase when the complement alternative pathway system is activated, which could lead to CFH being positively correlated with BMI and TG levels.

According to the criteria for defining obesity and hypertriglyceridemia in China [28, 29], the subjects were divided into three categories by BMI, and two categories by TG, as described in the results section above. Our results indicated that overweight or obese pregnant women, and women with hypertriglyceridemia, had significantly higher levels of CFH as compared with other categories, which provides a novel conceptual framework for determining the impact of overweight, obesity and hypertriglyceridemia on regulating the complement system.

Previous researches have reported that CFH elevated in pregnancy [19], and this study found that CFH level increased with the gestational age. Since CFH is a complement system inhibitor, it might be a mechanism of immunosuppression in pregnancy.

Moreover, we found that the CFH level had a moderately positive association with ALP, and ALP was an independent risk factor for serum CFH levels. As we know, ALP in pregnant mothers is mainly derived from placental tissues, the liver and bone [30, 31], and these tissues also affect the complement system and CFH expression [32–35]. This connection might account for the positive association between serum CFH and ALP.

This study also demonstrated that CFH was slightly (r < 0.3) associated with TB, age, and UA, and these factors might be independent risk factors for CFH levels in all subjects. The CFH level was slightly negatively associated with TB. Basiglio et al. reviewed that unconjugated bilirubin could inhibit activation of the complement system by preventing complement factor C1q interacting with immunoglobulins, and this might decrease CFH levels when the complement system was inhibited [36–38]. CFH was slightly positively correlated with age, which could be attributed to normal physiological phenomenon since previously published literature reported that the CFH level was significantly higher in adults than in neonates [39]. Previous work also similarly showed that UA was positively connected with complement C3 in adults, and that UA could stimulate the expression of complement C3 in a dose-dependent fashion [40]. Thus, the rising CFH levels might be a compensatory reaction after UA stimulated the complement system.

Some results of this study were not generally consistent with previous work conducted in Chinese females. Shen et al. used proteomic analysis and found that CFH changed significantly in GDM as compared with non-GDM controls at 12-16th gestational age after adjusting for maternal age, gravity, parity, BMI, gestational age at delivery and gestational age at time of sample collection [21]. However, our study found that there was no significant difference of CFH levels on comparing GDM and non-GDM after adjusting for other clinical characteristics. This discordance could be caused by the fact that the case numbers of the GDM group were relatively small and the detection methods of CFH were different.

In the Moreno-Navarrete et al. study, the CFH level was negatively associated with insulin sensitivity [16], therefore GDM patients with insulin resistance were speculated to have elevated CFH levels. Although our study found the CFH level was significantly higher in GDM than non-GDM controls, CFH was not independently related to GDM and avderse pregnancy outcomes. Therefore, it is rational to consider that although CFH is positive related with insulin resistance, it is not independent risk factors of insulin resistance. Insulin resistance is commonly exhibited in GDM, impaired glucose tolerance and T2DM, and these conditions are more likely to have high BMI and TG. In other words, it might be possible that the body adipose component and TG, but not the resulting CFH alterations, independently and directly influence insulin resistance in pregnancy.

This study had some limitations. First, the current study recruited a relatively small sample size of women with progressive GDM. Second, the lack of data reflecting islet β cell function such as fasting insulin and C-peptide levels resulted in the defect of the putative association between CFH and insulin resistance during pregnancy.

## Conclusion

This study helps advance our understanding of the role of CFH and the complement system in GDM and pregnancy. The data showed that the CFH level was positively associated with BMI, TG, gestational age, ALP, age and UA, and was negatively correlated with TB. These factors were independent risk factors for CFH levels which might affect the complement system activity when women are pregnant. However, CFH levels were not independently correlated with GDM and avderse pregnancy outcomes. Future studies of the associations between CFH and insulin resistance in pregnancy are indeed warranted.

## Supplementary Information

**Additional file 1.**

## Data Availability

The datasets generated and analyzed during the current reported work are available from the corresponding authors upon reasonable request.

## Abbreviations

CFH: Complement factor H
GDM: gestational diabetes mellitus
TG: total triglycerides
BMI: body mass index
PROM: premature rupture of membranes
CVD: cardiovascular disease
T2DM: type 2 diabetes mellitus
CRP: C-reactive protein
75-g OGTT: 75-g oral glucose tolerance test
GA: Glycated serum albumin
AST: aspartate aminotransferase
ALT: alanine aminotransferase
TB: total bilirubin
γ-GT: γ-glutamyltransferase
ALP: alkaline phosphatase
Cr: creatinine
BUN: blood urea nitrogen
UA: uric acid
TC: total cholesterol
LDL-C: low density lipoprotein cholesterol
ALB: albumin
BCG: Bromocresol Green
CV: coefficients of variation
OR: odds ratios
CIs: confidence intervals
SBP: systolic blood pressure
DBP: diastolic blood pressure
FPG: fasting plasma glucose
HbA1c: glycosylated hemoglobin A1c
IQR: Interquartile range
SD: standard deviation
