# Supplementary material for: The role of complement factor H in gestational diabetes mellitus and pregnancy
Source: BMC Pregnancy Childbirth. 2021 Aug 17;21:562. doi: 10.1186/s12884-021-04031-w (PMC8369714; doi:10.1186/s12884-021-04031-w)
Supplement: Supplementary file 1 — Additional file 1. [file 12884_2021_4031_MOESM1_ESM.pdf]

Additional file 1: Questionnaire for participant enrollment at the first prenatal visit

ID Number: ________________________	
Date at the first prenatal visit:	_______/_______/_____(year/month/day)
Gestational age:	_______/_______(week/day)

Part 1: Sociodemographic characteristics

Age:	_____ years
Height:	_____ cm
Weight:	_____. ___ kg
Pre-pregnancy weight:	_____. ___ kg
Body mass index (BMI):	_____. ___ kg/m^2
Pre-pregnancy BMI:	_____. ___ kg/m^2
Systolic blood pressure:	________ mmHg
Diastolic blood pressure:	________ mmHg
Place of Residence:	________________________

Part 2: Clinical characteristics

Date of the Last menstrual period:	_______/_______/_____(year/month/day)
Expected date of delivery:	_______/_______/_____(year/month/day)
Gravidity (number of pregnancies):	_______ time(s)
Parity (number of total births):	_______ time(s)
Miscarriage/abortion:	_______ time(s)
History of gestational diabetes mellitus：	①yes ②no
Family history of diabetes (first-degree relatives):	①yes ②no
